# Factors influencing the timeliness of care for patients with lung cancer in Bangladesh

**DOI:** 10.1186/s12913-023-09154-8

**Published:** 2023-03-16

**Authors:** Adnan Ansar, Virginia Lewis, Christine Faye McDonald, Chaojie Liu, Muhammad Aziz Rahman

**Affiliations:** 1 School of Nursing and Midwifery, College of Science Health and Engineering, La Trobe University, Kingsbury Drive, Bundoora, Melbourne, VIC 3086 Australia; 2 Institute for Breathing and Sleep (IBAS), Melbourne, Australia; 3 Australian Institute for Primary Care and Aging, La Trobe University, Melbourne, Australia; 4 Department of Respiratory & Sleep Medicine, Austin Health, Melbourne, Australia; 5 University of Melbourne, Melbourne, Australia; 6 School of Psychology and Public Health, La Trobe University, Melbourne, Australia; 7 School of Health, Federation University Australia, Berwick, Australia; 8 Department of Noncommunicable Diseases, Bangladesh University of Health Sciences (BUHS), Dhaka, Bangladesh; 9 Faculty of Public Health, Universitas Airlangga, Surabaya, Indonesia

**Keywords:** Delay, Timeliness, Intervals, Care seeking, Lung cancer, Bangladesh

## Abstract

**Background:**

This study explored the factors associated with timeliness of care in the healthcare seeking pathway among patients with lung cancer in Bangladesh.

**Methods:**

A structured questionnaire was used for data collection from 418 patients with lung cancer through face-to-face interviews in three tertiary care hospitals. Log-rank tests were performed to test differences in the length of intervals between points in healthcare by socioeconomic characteristics and care seeking behaviours of the patients. Cox Proportional Hazard (PH) regression analysis was performed to identify the predictors of the intervals after adjustment for variations in other variables.

**Results:**

A higher education level was associated significantly (p < 0.05) with a shorter interval between first contact with a healthcare provider (HCP) and diagnosis (median 81 days) and initiation of treatment (median 101 days). Higher monthly household income was associated significantly with a shorter time from first contact and diagnosis (median 91 days), onset of symptom and diagnosis (median 99 days), onset of symptom and treatment (median 122 days), and first contact with any HCP to treatment (median 111 days). Consulting with additional HCPs prior to diagnosis was associated significantly with longer intervals from first contact with any HCP and diagnosis (median 127 days), onset of symptom and diagnosis (median 154 days), onset of symptom and treatment (median 205 days), and first contact with any HCP to treatment (median 174 days). Consulting with informal HCPs was associated significantly with a longer time interval from symptom to treatment (median 171 days). Having more than one triggering symptom was associated significantly with a shorter interval between onset of symptoms and first contact with any HCP.

**Conclusion:**

The predictors for timeliness of lung cancer care used in this study affected different intervals in the care seeking pathway. Higher education and income predicted shorter intervals whereas consulting informal healthcare providers and multiple providers were associated with longer intervals.

**Supplementary Information:**

The online version contains supplementary material available at 10.1186/s12913-023-09154-8.

## Introduction

Globally, lung cancer is the most common type of malignancy with high incidence and mortality [1]. There were 2.1 million new cases and 1.8 million deaths from lung cancer during 2018 [2]. Lung cancer accounts for 11.6% of all diagnosed cancers and almost 1 in 5 of all cancer deaths [3]. The incidence of cancer in general is higher in high income countries (HICs) compared to low and middle income countries (LMICs), but cancer mortality is highest in LMICs [4]. It is projected that by 2030, 75% of all cancer deaths globally will be in LMICs [5]. Even though lung cancer incidence and mortality vary across countries, the 5-year survival rates are only 10–20% across the globe as the majority are diagnosed at an advanced stage of the disease [2]. Hence, timely diagnosis and treatment is crucial to ensure a better prognosis and survival for patients with lung cancer [6, 7].

A scoping review by Malalasekera et al. (2018) identified 78 factors associated with delays in lung cancer care, although causality was not established. All but one of the studies reported were from HICs [8]. Late stage at presentation, lower levels of education or socioeconomic position, and lack of early clinical symptoms were identified as the most frequent patient-related factors associated with delay in seeking care. In addition, health system factors such as ambiguous clinical features/symptoms, long waiting times for initial assessment, and late initiation of treatments were also found to be important [8]. Even though general factors influencing timeliness of care are similar between HICs and LMICs, additional factors contributing to delays in care in LMICs have been identified. Late stage diagnosis of lung cancer in LMICs is attributed to lack of awareness, inaccessibility of healthcare, lack of infrastructure and equipment, ineffective referral mechanisms, inadequate public funding of health services, and unaffordable treatment [9, 10]. Other contextual factors that vary between LMICs and HICs include the prevalence and type of health conditions that may delay a lung cancer diagnosis. For example, rates of tuberculosis are high in LMICs; with symptoms closely mimicking those of lung cancer [11–14]. Multiple visits to primary healthcare providers increase delays in diagnosis and treatment [13, 15–17], which may be accounted for by the inadequacy of primary care, lack of adequate specialist care, lack of an appropriate referral system and lack of coordination of care. It is recognised that seeking care from informal healthcare providers such as untrained or under-qualified local healthcare providers, herbal medicine and traditional healing practitioners occur more commonly in pluralistic health care systems, with consequent delays in the institution of effective evidence-based medical care [18–21].

Numerous studies have measured the duration of intervals from onset of symptoms to seeking care from a qualified healthcare provider [9, 17], to diagnosis [22, 23] and to treatment [22, 24–26] of lung cancer, with each of these intervals measured separately. Lack of symptoms, poor awareness of symptoms, older age, delay in referral, lack of referral guidelines, multiple consultations and, delay in initiation of any type of treatment have been identified as factors affecting the duration of intervals [8]. However, there is a paucity of research exploring factors associated with durations of segmented intervals in the care seeking pathway from the perspective of LMICs. Understanding the factors influencing delays in different intervals along the patient care pathway can provide insight into the local health system and provide evidence that can assist in developing targeted interventions for system improvement.

Lung cancer is the most commonly diagnosed cancer in Bangladesh [27], and the majority of cases are diagnosed at an advanced stage of the disease [28]. The objective of our study was to explore factors associated with timeliness of care in the healthcare seeking pathway among patients with lung cancer in Bangladesh. The following research questions were asked to address the objective.

1. What commonly reported factors were associated with the duration of length of different intervals in the care seeking pathway among patients with lung cancer in Bangladesh?
2. Was there any difference in timeliness of cancer care between healthcare utilization through formal and informal providers?

## Methods

The study design and data collection procedures of this study are briefly described below, and a detailed description has been published [29].

### Study design and population

Data were collected about patients with lung cancer between 10 and 2019 and 13 February 2020 in three tertiary hospitals in the capital city of Dhaka, Bangladesh which included National Institute of Cancer Research and Hospital (NICRH), Bangabandhu Sheikh Mujib Medical University (BSMMU), and Ahsania Mission Cancer & General Hospital (AMCGH).

Participants included admitted patients in the study hospitals, aged ≥ 18 years with a diagnosis of lung cancer during the period of data collection. Participants could also nominate a family member or carer (if needed), to provide information on their behalf. Potential participants were identified by the healthcare providers at the hospitals as per the inclusion criteria and were invited to participate in the study. Informed written consent was obtained from all study participants. In the case of illiterate participants, a thumb print was provided as an indication of informed consent. Of 424 eligible patients identified, only six declined to participate and 418 complete questionnaires were returned, which is large enough to detect an effect size of 0.5 (small to medium) for a regression modelling with 20 predictors whilst α being set at 0.05 and statistical power (1-β) being set at 0.8 [30].

### Questionnaire and data collection procedure

Data were collected using a pre-tested, bespoke, structured questionnaire developed by the authors and informed by relevant literature [31, 32] and national surveys. The questionnaire was in Bengali which is the local native language. It had four sections including (1) socio-demographics, (2) history of illness (3) symptom history, and (4) description of help-seeking. The questionnaire was administered face-to-face by three data collectors. After obtaining written informed consent, interviews were conducted at the bedside (while maintaining privacy), or, in the case of the caregiver, the interview was conducted in the duty doctor’s room in private. To minimize recall bias, all dates that were provided by the participants were cross checked and verified through reviewing patient-held and hospital clinical files. In case of a mismatch between information provided by the participant and hospital documents; the dates derived from the medical record were accepted.

### Variables and measures

**Timepoints**: Five timepoints commonly reported in the literature [33] were measured: (i) date of patient reported onset of symptom(s), (ii) date of first contact with any healthcare provider, (iii) date of diagnosis, (iv) date of referral for treatment, and (v) date of initiation of treatment.

**Time Intervals**: Time intervals were estimated by calculating the duration (days) from one time point to another. The time interval was recorded as ‘0’ days if the starting and end events occurred on the same day (e.g., participant contacted a healthcare provider on the first day the symptoms appeared, diagnosed on the day of first healthcare provider contact, referred for treatment at the time of receiving the diagnosis and started treatment on the day of referral). Eight time intervals in the lung cancer care pathway in common use identified through a scoping review (under review) were calculated. All the intervals are measured in days.

- Onset of symptom(s) to first contact with any healthcare provider, including informal providers.
- First contact with any healthcare provider to diagnosis.
- Diagnosis to referral for treatment.
- Referral for treatment to initiation of treatment.
- Onset of symptom(s) to diagnosis.
- Diagnosis to initiation of treatment.
- Onset of symptom(s) to initiation of treatment.
- First contact with any healthcare provider to initiation of treatment.

### Data analysis

#### Justification for choice of independent variables

The behavioral model of health service utilization developed by Andersen emphasises demographic characteristics (age, sex, marital status), socio-structural characteristics (education level, race, ethnicity, family size), health beliefs (values, attitudes), family characteristics (income, insurance coverage), community characteristics (availability of resources, proximity to care, region of country), and need factors (individual experience of symptoms, health status) as determinants or predictors of health seeking behavior [34–37]. Guided by this concept, predictors relevant to the objective and capacity of the study were selected for use in regression models.

This study explored whether locally relevant socio-demographic characteristics (age, sex, marital status, family type, urban/rural residence, education, household income), number of triggering symptoms, type of first healthcare provider, time to travel to first healthcare provider, whether or not an additional provider was consulted before diagnosis, and time to travel to a diagnostic facility were associated with and/or predicted a longer or shorter duration of intervals along the care-seeking pathway. Categories for each variable are displayed in Table 2. In Bangladesh primary education is from year one to five, secondary education is year six to ten and higher education is from year ten to twelve. Urban/rural residency was determined based on patient residential municipality status, while data on household income was acquired by asking the participant the total income of every wage earner and other source of income in their household. A selected number of predictive variables were chosen because of concern about the ability of people to participate in the study, given they were hospitalised with their illness and a long survey could impose distress.

#### Regression models

Variables considered logically and temporally relevant to an interval were included. In other words, some variables were not used as predictors for some intervals. For example, the interval from first symptom to first contact included age, sex, marital status, education, urban/rural residence, income, family type, number of triggering symptoms, type of first healthcare provider and time to travel to healthcare provider, but not whether or not an additional healthcare provider was consulted before diagnosis or time to travel to a diagnostic facility, as these two were not relevant to that interval.

In the univariate analysis of the time intervals, we measured the association between the intervals and the factors of interest by comparing the median length of intervals within different levels of a factor by means of the log-rank test. Table 1 shows the initial proposed regression models with selected predictor variables. Afterwards, we performed Cox Proportional Hazard (PH) regression analyses to assess the association of our set of independent variables with the intervals of interest. Detailed steps of Cox PH regression models are provided in the additional file 1.

**Table 1:** Proposed regressions for each interval

Time intervals	Initial proposed models
Duration between onset of symptom(s) and first contact with any healthcare provider	Age + Sex + Marital status + Education + Urban/rural + HH income + Type of family + Number of triggering symptoms to seek HCP + Type of first HCP for first contact + Time to travel to HCP
Duration between first contact with any healthcare provider and diagnosis	Age + Sex + Marital status + Education + Urban/rural + HH income + Type of family + Number of triggering symptoms to seek HCP + Type of first HCP for first contact + Time to travel to HCP + Number of additional HCP before diagnosis + Time to travel to Diagnostic facility
Duration between diagnosis and referral	Age + Sex + Marital status + Education + Urban/rural + HH income + Type of family
Duration between referral and initiation of treatment	Age + Sex + Marital status + Education + Urban/rural + HH income + Type of family
Duration between onset of symptom(s) and diagnosis	Age + Sex + Marital status + Education + Urban/rural + HH income + Type of family + Number of triggering symptoms to seek HCP + Type of first HCP for first contact + Time to travel to HCP + Number of additional HCP before diagnosis + Time to travel to Diagnostic facility
Duration between diagnosis and initiation of treatment	Age + Sex + Marital status + Education + Urban/rural + HH income + Type of family + Number of triggering symptoms to seek HCP + Type of first HCP for first contact + Time to travel to HCP + Number of additional HCP before diagnosis + Time to travel to Diagnostic facility
Total duration from onset of symptom(s) to initiation of treatment	Age + Sex + Marital status + Education + Urban/rural + HH income + Type of family + Number of triggering symptoms to seek HCP + Type of first HCP for first contact + Time to travel to HCP + Number of additional HCP before diagnosis + Time to travel to Diagnostic facility
Duration between first contact with any healthcare provider to treatment initiation	Age + Sex + Marital status + Education + Urban/rural + HH income + Type of family + Type of first HCP for first contact + Time to travel to HCP + Number of additional HCP before diagnosis + Time to travel to Diagnostic facility

## Results

The mean age of the participants was 57 years (SD ± 9.86, range 25–82 years). The majority of patients were over 55 years old (68%), with less than 10% aged under 45 years. Participants were predominantly from rural areas (88%) and the majority of participants were illiterate (64%). The mean number of years of education was 4 years (SD ± 5,range 0–17 years). The median monthly household income was Bangladeshi Taka (BDT) 15,500 (USD 182). The lowest quartile had an income of BDT 10,000 (US$ 118) and below, whereas the upper quartile had an income of BDT 100,000 (US$ 1176) and above. Table 2 presents the results of the univariate analysis (crude hazard ratio) of the time intervals.

### Symptom(s) to first contact with any healthcare provider

Higher education level (median 15 days, p < 0.05) and longer travel time to a healthcare provider (median 14 days, p < 0.05) were significantly associated with longer duration from onset of symptoms to first contact with any healthcare provider, whereas having first contact with an informal healthcare provider (median 10 days, p < 0.05) and a greater number of triggering symptoms (median 9 days, p < 0.05) were associated with shorter duration for the same interval.

### First contact with any healthcare provider to diagnosis and Symptom(s) to diagnosis

Higher education level (median 81 & 95 days, p < 0.05), higher monthly household income (median 91 & 99 days, p < 0.05), and longer travel time to the first healthcare provider (median 92 & 113 days, p < 0.05) were significantly associated with shorter duration of interval for both of these intervals, whereas, having contact with an informal healthcare provider (median 121 & 136 days, p < 0.05), a greater number of triggering symptoms (median 136 & 150 days, p < 0.05) and consulting with more than three additional healthcare providers prior to diagnosis (median 127 & 154 days, p < 0.05) were associated with longer duration of the interval.

### Diagnosis to initiation of treatment

In the case of the interval from diagnosis to treatment, having first contact with an informal healthcare provider (median 25 days, p < 0.05) was significantly associated with a longer duration.

### Symptom to initiation of treatment

Higher education level (median 117 days, p < 0.05), higher monthly household income (BDT 50,001–100,000) (median 122, p < 0.05) and longer travel time to first healthcare provider (median 140 days, p < 0.05) were significantly associated with a shorter duration of the interval from symptom to treatment, whereas first contact with an informal healthcare provider (median 171 days, p < 0.05), a greater number of triggering symptoms (median 191 days, p < 0.05) and pre-diagnostic consultation with more than three healthcare providers (median 205, p < 0.05) were associated with longer duration of the interval.

### First contact with any healthcare provider to initiation of treatment

Higher education level (median 101, p < 0.05), higher monthly income (BDT 50,001–100,000) (median 111 days, p < 0.05), and longer travel time to first healthcare provider (median 125 days, p < 0.05) were significantly associated with shorter duration of interval from first contact with any healthcare provider to treatment. Whereas having an informal healthcare provider as first contact (median 156 days, p < 0.05), a greater number of triggering symptoms (median 180, p < 0.05) and pre-diagnostic consultation with more than three healthcare providers (median 174 days, p < 0.05) were associated with longer duration of the interval.

We did not find any significant variables for the ‘Duration between referral and treatment start ‘ and ‘Duration between referral and initiation of treatment’. Socio-demographic characteristics of the respondents such as age, sex, marital status, urban/rural location of residence, family structure was not significantly associated with the intervals of interest in the univariate analysis (Table 2).

**Table 2:** Association between participant characteristics and Intervals (univariate analysis/unadjusted/crude hazard ratio)

	Sample Size	Symptom(s) – First contact		First contact - Diagnosis		Symptom(s) - Diagnosis		Diagnosis – Treatment		Symptom(s) – Treatment		First contact – Treatment	
	(n, %)	Median days (25–75% IQR)	p-value	Median days (25–75% IQR)	p-value	Median days (25–75% IQR)	p-value	Median days (25–75% IQR)	p-value	Median days (25–75% IQR)	p-value	Median days (25–75% IQR)	p-value
**Age Group (in years)**													
< 40	20 (4.8)	9 (2, 15)	0.76	83 (47, 129)	0.26	85 (56, 131)		27 (22, 41)	0.20	120 (81, 223)	0.06	108 (70, 221)	0.06
41–49	60 (14.4)	10 (7, 16)		93 (53, 154)		104 (67, 169)		25 (12, 45)		132 (97, 205)		120 (94, 180)	
50–59	128 (30.6)	10 (5, 15)		108 (58, 162)		123 (76, 183)	0.22	24 (13, 50)		153 (101, 235)		140 (90, 210)	
60–69	161 (38.5)	10 (6, 16)		113 (72, 191)		127 (81, 212)		20 (12, 47)		161 (109, 266)		146 (95, 244)	
≥ 70	49 (11.7)	15 (7, 16)		106 (56, 168)		122 (64, 190)		18 (9, 35)		153 (106, 207)		130 (96, 193)	
**Sex**													
Male	375 (89.7)	10 (6, 15)	0.86	108 (61, 175)	0.26	122 (76, 193)	0.2	22 (12, 47)	0.16	151 (103, 234)	0.48	135 (91, 212)	0.53
Female	43 (10.3)	10 (7, 16)		93 (52, 159)		105 (63, 174)		26 (10, 68)		140 (93, 220)		132 (89, 203)	
**Marital Status**													
Married	388 (92.8)	10 (6, 16)	0.53	107 (58, 169)	0.36	121 (74, 190)	0.35	23 (12, 47)	0.6	151 (101, 224)	0.42	135 (91, 209)	0.42
Not Married	30 (7.2)	10 (7, 15)		105 (69, 197)		125 (77, 224)		15 (9, 48)		137 (108, 271)		132 (100, 256)	
**Location of Residence**													
Urban	50 (12)	10 (6, 16)	0.53	79 (44, 126)	0.09	91 (60, 162)	0.2	18 (9, 40)	0.25	116 (85, 182)	0.18	99 (60, 165)	0.07
Rural	368 (88)	10 (6, 16)		111 (63, 177)		123 (76, 195)		24 (12, 48)		156 (107, 234)		142 (95, 217)	
**Family Structure**													
Nuclear family	164 (39.2)	10 (7, 15)	0.96	93 (54, 174)	0.67	107 (69, 78)	0.67	20 (11, 46)	0.54	137 (99, 238)	0.75	125 (89, 210)	0.71
Joint/extended family	254 (60.8)	10 (6, 16)		114 (65, 170)		127 (190, 192)		25 (13, 47)		159 (108, 224)		146 (95, 210)	
**Highest Education Level (years)**													
Illiterate & primary education (≤ 5)	297 (71.1)	10 (6, 15)	**0.04**	118 (69, 182)	**< 0.01**	130 (81, 197		22 (12, 47)	0.11	162 (111, 240)	**< 0.01**	153 (99, 227)	**< 0.01**
Secondary education (6–10)	73 (17.5)	10 (6, 14)		92 (45, 159)		106 (58, 190	**< 0.01**	25 (13, 51)		144 (91, 222)		129 (82, 209)	
≥Higher secondary education (≥ 11)	48 (11.5)	15 (7, 16)		81 (52, 108)		95 (62, 127)		20 (9, 40)		117 (91, 149)		101 (75, 125)	
**Monthly Household Income**													
BDT ≤ 15,000 (≤$176)	209 (50)	10 (6, 15)	0.36	124 (56, 197)	**< 0.01**	139 (71, 224)	**< 0.01**	25 (14, 48)	0.36	169 (107, 252)	**< 0.01**	160 (93, 242)	**< 0.01**
BDT 15,001–50,000 ($176–588)	126 (30.1)	10 (5, 16)		109 (70, 178)		122 (82, 193)		21 (12, 47)		146 (107, 238)		136 (95, 230)	
BDT 50,001–100,000 ($588–1176)	51 (12.2)	15 (8, 16)		86 (56, 115)		101 (72, 125)		13 (8, 47)		122 (91, 175)		111 (76, 156)	
BDT ≥ 100,001 (≥$1176)	32 (7.7)	15 (7, 16)		91 (53, 123)		99 (70, 133)		12 (8, 46)		133 (89, 180)		116 (80, 161)	
**Travel time to first provider**													
< 30 min	302 (72.2)	10 (6, 15)	**0.01**	109 (64, 187)	**< 0.01**	123 (77, 198)	**< 0.01**	22 (12, 47)	0.47	153 (106, 242)	**0.01**	136 (96, 224)	**< 0.01**
≥ 31 min	116 (27.8)	14 (6, 20)		92 (45, 153)		113 (62, 166)		25 (11, 48)		140 (91, 198)		125 (78, 179)	
**First healthcare provider**													
Formal healthcare provider	168 (40.2)	13 (6, 16)	**0.02**	89 (45, 142)	**< 0.01**	104 (59, 157)	**< 0.01**	19 (9, 41)	**< 0.01**	130 (85, 195)	**< 0.01**	117 (72, 173)	**< 0.01**
Informal healthcare provider	250 (59.8)	10 (6, 15)		121 (75, 197)		136 (85, 212)		25 (14, 51)		171 (113, 266)		156 (102, 242)	
**Triggering symptoms**													
One triggering symptom	226 (54.1)	12 (7, 16)	**0.01**	95 (56, 156)	**< 0.01**	110 (76, 174)		22 (11, 48)	0.83	139 (102, 210)	**0.01**	126 (93, 187)	**< 0.01**
Two triggering symptoms	144 (34.4)	10 (5, 15)		116 (58, 192)		122 (71, 224)	**< 0.01**	23 (13, 44)		154 (100, 250)		145 (91, 232)	
≥Three triggering symptoms	48 (11.5)	9 (5, 13)		136 (67, 244)		150 (82, 258)		21 (11, 47)		191 (108, 290)		180 (106, 274)	
**Additional HCP pre-diagnosis**													
One healthcare provider	105 (27.4)	10 (6, 15)		93 (43, 159)		103 (62, 173)		18 (10, 41)		130 (93, 199)		120 (87, 178)	
Two healthcare providers	176 (46.0)	10 (6, 16)	0.80	115 (74, 169)	**< 0.01**	129 (84, 187)	**< 0.01**	22 (11, 48)	0.09	159 (111, 233)	**< 0.01**	143 (98, 221)	**< 0.01**
≥Three healthcare providers	102 (26.6)	10 (6, 15)		127 (79, 232)		154 (91, 242)		27 (14, 52)		205 (123, 289)		174 (110, 274)	
**Travel time to diagnostic facility**													
≤ 60 min	42 (10)	10 (7, 16)	0.86	102 (47, 166)	0.51	121 (68, 183)	0.51	19 (11, 32)	0.21	135 (90, 212)	0.27	122 (80, 202)	0.30
≥ 61 min	374 (89.5)	10 (6, 16)		107 (61, 174)		121 (74, 192)		23 (12, 47)		151 (103, 233)		135 (91, 212)	
**Total**	**418 (100)**	**10 (6, 16)**		**107 (60, 174)**		**121 (74, 192)**		**23 (12, 47)**		**151 (101, 233)**		**135 (91, 212)**	

*The median and percentiles were estimated using Kaplan-Meier nonparametric estimation method. The p-values were obtained from the log-rank test of equality of survival functions between two or more independent group

**Fig. 1:**
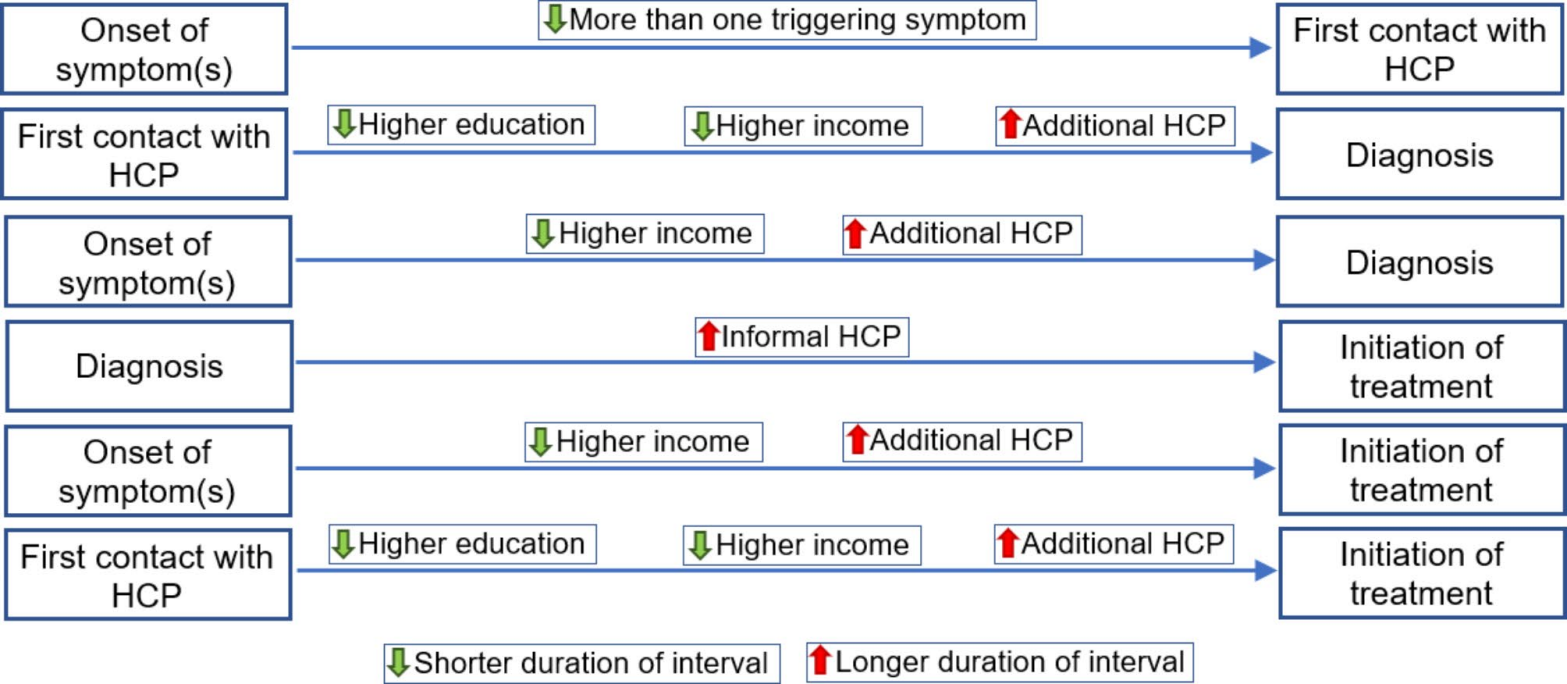
Factors influencing the duration of segmented intervals

Table 3 demonstrates the results of the Cox PH regression analysis for the intervals under this study and Fig. 1 illustrates the factors influencing the each of the intervals in the disease care pathway.

### Symptom(s) – first contact with any healthcare provider

Only the number of triggering symptoms (p < 0.05) was associated significantly with the time span from symptom to first contact with any healthcare provider. Compared to patients with only one triggering symptom (reference group), patients with two and three or more triggering symptoms had adjusted hazard ratios greater than one (HR > 1). That is, a higher number of triggering symptoms is associated significantly with shorter intervals between symptom and first contact with any healthcare provider.

**First contact with any healthcare provider – Diagnosis**: Educational level (p < 0.05), monthly household income (overall p < 0.05), and number of additional healthcare providers before diagnosis (p < 0.05) were significantly associated with the first contact-diagnosis interval. The patient group with secondary education or above had a significantly shorter interval from first contact to diagnosis than (adjusted HR = 1.58; 95% CI: 1.12–2.25) the group with primary or no education (reference group). Compared to the patient group with a monthly income less than 15,000 Bangladeshi Taka (BDT) (reference group), both patient groups with monthly income between 50,000 to 1,00,000 and greater than 1,00,000 BDT had a significantly shorter (adjusted HR > 1) first contact-diagnosis interval. Patients with two healthcare providers or three or more healthcare providers had longer intervals (adjusted HR < 1) compared to the group with only one healthcare provider (reference group).

### Symptom(s) – diagnosis

There was a significant association of the onset of symptom-diagnosis interval with monthly household income (p < 0.05) and number of additional healthcare providers (p < 0.05). Monthly household incomes between 50,000 BDT to 1,00,000 BDT and above 1,00,000 BDT (comparison group) are significantly associated with shorter symptom to diagnosis interval (adjusted HR > 1) compared to the reference group with monthly income less than 15,000 BDT (reference group). On the other hand, consulting with two healthcare providers and three or more healthcare providers (comparison group) was associated significantly with a longer onset of symptom(s) to diagnosis interval (adjusted HR < 1).

### Diagnosis – initiation of treatment

There was a significant association of the time span from diagnosis to treatment only with the type of first healthcare provider (p < 0.05). Patients who consulted with informal healthcare providers (comparison group) had longer duration of diagnosis to initiation of treatment interval (adjusted HR < 1), compared to those who consulted with formal healthcare providers (reference group).

### Symptom(s) – initiation of treatment

In the case of the interval between onset of symptom to initiation of treatment, monthly household income (p < 0.05) and the number of additional healthcare providers consulted before diagnosis (p < 0.05) were significantly associated with the length of this interval. In comparison with the patient group with a monthly income less than BDT 15,000 (reference group), both of the patient groups with monthly incomes between BDT 50,000 to 1,00,000 and greater than BDT 100,000 had a significantly shorter (adjusted HR > 1) first contact-diagnosis interval. That is, increased monthly household income was associated with shorter onset of symptom to initiation of treatment intervals. On the contrary, seeking care from additional healthcare providers (two HCP and three or more HCP – comparison group) was associated significantly with longer symptom-treatment intervals (adjusted HR < 1) in compare to the reference group of seeking care from one healthcare provider.

**First contact with any healthcare provider – Initiation of treatment**: Similar to the first contact-diagnosis interval, the time span from first contact to treatment initiation was significantly associated with highest education level (p < 0.05), monthly household income (p < 0.05), and number of additional healthcare provider consulted before diagnosis (p < 0.05). The patient group with higher secondary education or above differed significantly (adjusted HR = 1.66; 95% CI: 1.16–2.66) from the group with primary or no education (reference group) and, the interval from first contact with any healthcare provider to initiation of treatment was significantly shorter (adjusted HR > 1) than the reference group. Compared to patients with monthly income less than 15,000 BDT (reference group), patients with monthly income between BDT 50,000 to 1,00,000 and greater than BDT 100,000 had a significantly shorter (adjusted HR > 1) first contact to initiation of treatment interval. On the other hand, the number of additional HCP consultation before diagnosis was significantly associated with longer first contact-diagnosis intervals. Patients who consulted two healthcare providers and three or more healthcare providers additionally (comparison group) had adjusted hazard ratios, HR < 1 (longer intervals) compared to the baseline group with only one healthcare provider consultation.

The association between the time intervals and the factors of interest can be summarized as follows:

- More than one triggering symptom was significantly associated with shorter intervals between onset of symptoms to first contact with any healthcare provider.
- Secondary school or above education level was significantly associated with a shorter interval between first contact with a healthcare provider to diagnosis and between first contact with a healthcare provider to initiation of treatment.
- Higher monthly household income was significantly associated with shorter first contact with a healthcare provider to diagnosis, onset of symptom to diagnosis, onset of symptom to initiation of treatment, and first contact with any healthcare provider to initiation of treatment intervals.
- Consulting with informal healthcare providers was significantly associated with longer duration of the diagnosis to initiation of treatment interval.
- Consulting with additional HCP (healthcare providers) before diagnosis was significantly associated with longer first contact with any healthcare provider to diagnosis (three or more HCPs), onset of symptom to diagnosis (two HCPs, and three or more HCPs), onset of symptom to initiation of treatment (two HCPs, and three or more HCPs), and first contact with any healthcare provider to initiation of treatment intervals (three or more HCPs).

**Table 3:** Association between participant characteristics and intervals – Cox proportional hazard regression analysis (multivariable/adjusted)

Interval	Factor	Adjusted Hazard Ratio	95% CI	P-value
**Symptom(s) – First contact with any healthcare provider**	**Symptoms triggered to seek an HCP**			
	One triggering symptom	Reference		
	Two triggering symptoms	1.25	1.01–1.54	**0.04**
	Three or more triggering symptoms	1.44	1.05–1.97	**0.02**
**First contact with any healthcare provider – Diagnosis**	**Highest Education Level (years)**			
	Illiterate & primary education (≤ 5)	Reference		
	Secondary education (6–10)	0.98	0.74–1.29	0.86
	≥Higher secondary education (≥ 11)	1.58	1.12–2.25	**0.01**
	**Monthly Household Income**			
	BDT ≤ 15,000 (≤$176)	Reference		
	BDT 15,001–50,000 ($176–588)	1.08	0.85–1.37	0.54
	BDT 50,001–100,000 ($588–1176)	1.95	1.38–2.77	**< 0.01**
	BDT ≥ 100,001 (≥$1176)	1.72	1.13–2.61	**0.01**
	**Additional HCP pre-diagnosis**			
	One healthcare provider	Reference		
	Two healthcare providers	0.79	0.62–1.01	0.06
	Three or more healthcare providers	0.65	0.49–0.86	**< 0.01**
**Symptom(s) – Diagnosis**	**Monthly Household Income**			
	BDT ≤ 15,000 (≤$176)	Reference		
	BDT 15,001–50,000 ($176–588)	1.11	0.87–1.40	0.4
	BDT 50,001–100,000 ($588–1176)	2.1	1.52–2.91	**< 0.01**
	BDT ≥ 100,001 (≥$1176)	1.88	1.26–2.80	**< 0.01**
	**Additional HCP pre-diagnosis**			
	One healthcare provider	Reference		
	Two healthcare providers	0.78	0.61–0.99	**0.04**
	Three or more healthcare provider	0.63	0.48–0.84	**< 0.01**
**Diagnosis – Treatment**	**First healthcare provider**			
	Formal healthcare provider	Reference		
	Informal healthcare provider	0.77	0.63–0.94	**0.01**
**Symptom(s) – Treatment**	**Monthly Household Income**			
	BDT ≤ 15,000 (≤$176)	Reference		
	BDT 15,001–50,000 ($176–588)	1.09	0.86–1.37	0.68
	BDT 50,001–100,000 ($588–1176)	2.05	1.48–2.82	**< 0.01**
	BDT ≥ 100,001 (≥$1176)	1.94	1.30–2.89	**< 0.01**
	**Additional HCP pre-diagnosis**			
	One healthcare provider	Reference		
	Two healthcare providers	0.77	0.60–0.98	**0.04**
	Three or more healthcare providers	0.6	0.46–0.80	**< 0.01**
**First Contact with any healthcare provider – Treatment**	**Highest Education Level (years)**			
	Illiterate & primary education (≤ 5)	Reference		
	Secondary education (6–10)	0.92	0.70–1.22	0.58
	≥Higher secondary education (≥ 11)	1.66	1.16–2.36	**0.01**
	**Monthly Household Income**			
	BDT ≤ 15,000 (≤$176)	Reference		
	BDT 15,001–50,000 ($176–588)	1.05	0.82–1.33	0.7
	BDT 50,001–100,000 ($588–1176)	1.84	1.30–2.61	**< 0.01**
	BDT ≥ 100,001 (≥$1176)	1.71	1.13–2.60	**0.01**
	**Additional HCP pre-diagnosis**			
	One healthcare provider	Reference		
	Two healthcare providers	0.79	0.62–1.01	0.06
	Three or more healthcare providers	0.61	0.46–0.81	**< 0.01**

* In Cox PH regression analysis, an adjusted hazard ratio, HR > 1 corresponds to a shorter time span or length of the interval. Similarly, HR < 1 indicates longer intervals

## Discussion

To the best of our knowledge, this is the first study which has provided evidence of factors predicting delay in seeking care for patients with lung cancer in Bangladesh. We explored six intervals in the care pathway and found that some factors affected multiple intervals while others only affected one. The factors that were identified as predictors for delay in our study are the presence of multiple triggering symptoms, low level of education, low household income, consulting multiple providers prior to diagnosis, and seeking care from informal health care providers.

The health system in Bangladesh is different from those in western countries. For example, there is no compulsory requirement for a patient to have a formal referral to access different providers, and there is no mechanism to know whether patients have taken up referrals [38]. In Bangladesh, the public sector provides not-for-profit services that include preventive, curative, promotive and rehabilitative. In contrast, the private sector providers are mostly for-profit curative services that operate in urban areas, mainly in the capital city of Dhaka and other major cities. The private sector employs more providers than the public sector and many doctors who already employed in public sector are also employed in private sector. Many health professionals hold two jobs [38]. Pharmacies, traditional healers (ayurvedic, homeopathic, unanie/kabiraji - herbal medicine providers) and village doctors are the most common informal healthcare providers in Bangladesh [39]. Pharmacies, which might be more accurately described as retail medicine shops or drug sellers, are often the first point of contact for people in Bangladesh, particularly for those of lower socioeconomic status [40]. Although there is acknowledgement of a significant cancer burden in Bangladesh, reliable data are difficult to obtain, and no national cancer registry is maintained. As a result, articles and reports about cancer incidence and prevalence in Bangladesh are scarce [38].

Our findings suggested that a higher educational level was associated significantly with a shorter interval from ‘first contact with a healthcare provider to diagnosis and initiation of treatment’. A similar result was found in an Indian study [41] and in a review on LMICs by Lubuzo et al. (2020) [42]. The population in Bangladesh has a low literacy level which contributes to low health literacy. As a result, promoting behavioral change to encourage early help seeking for cancer through health education campaigns could be a challenge [38]. However, research conducted in Thailand suggested education level may not be a factor in the interval from onset of symptoms to first contact with a provider in countries that have universal health care schemes [43].

Having a higher monthly household income was associated significantly with a shorter interval from ‘onset of symptom to diagnosis’, ‘onset of symptom to treatment’ and ‘first contact with a healthcare provider to ‘diagnosis and initiation of treatment’ in our study. This finding was similar to two studies from India [31, 44] which found that higher household income was associated with shorter duration from onset of symptom to diagnosis. Studies from western countries have reported mixed findings about household income as a predictor for timely care [45, 46]. We did not find that income level was associated with the duration of the interval from ‘onset of symptom’ to ‘first contact with any provider’. This could be because initial contact was with an informal healthcare provider for almost two-thirds of the participants in our study; noting that these types of healthcare providers are more available and cheaper to access in Bangladesh [38].

Unlike other studies from developed countries [47–50] we did not find age, sex or marital status to be associated with delay at any interval in the disease care pathway. Living in an urban versus rural area was not associated with any delay in our study, whereas a study in South India found rurality is associated with timeliness of treatment initiation [51]. The proportion of respondents from urban areas in our study was small, so the power to find this difference was limited. We found that having three or more triggering symptoms compared to two or fewer was associated significantly with a shorter time interval between symptom onset and first contact with any healthcare provider. This is consistent with the findings of studies from UK [52, 53], and is related to a higher perceived need for healthcare driving health behaviour.

Seeking care from an informal healthcare provider was associated significantly with a longer duration in the interval from diagnosis to initiation of treatment in our study, but not with any other intervals. Findings from a single centre study on several cancers in India by Tiwari et al. (2015), found that seeking care from unqualified local practitioners and use of alternative medication caused delay in presentation to a medical facility [41]. Sachdeva et al. (2014), in a small single centre study from Haryana, north India, found that delay from onset of symptom to diagnosis can be attributable to the use of home remedies and seeking care from informal healthcare providers [44].

Consulting with multiple healthcare providers before diagnosis was associated significantly with delays in the onset of symptom to diagnosis and initiation of treatment, which is consistent with findings from other similar studies [13, 15–17, 31]. Our study also found consulting with multiple healthcare providers was associated significantly with longer intervals to diagnosis and to initiation of treatment from ‘first contact with any healthcare provider’. We did not collect information about the nature of the different providers when multiple providers were consulted.

Our study demonstrated intervals for lung cancer care were associated with Andersen’s socio-structural characteristics (education level) and family characteristics (household income) but not demographic characteristics (such as urban/rural residency) at different points in the care pathway. Experience of symptom(s) as a cue to seek healthcare was a significant predictor in this study as suggested by Andersen’s model [54, 55]. Seeking care from informal healthcare providers was a significant predictor for longer duration in this study; organisational factors described as contextual characteristics in Anderson’s model, such as the amount and distribution of services and personnel and the location of services, might have influenced choice of provider. Higher household income predicted shorter intervals in four intervals in the disease care pathway, consistent with Andersen’s ‘Behavior model of health service use’ which describes community per capita income and price of health services as important predictors for seeking healthcare [54, 55]. The fact that education and income levels were major predictors for timely care in most of the intervals indicates there are socioeconomic inequalities in access to healthcare for lung cancer in Bangladesh. In order to reduce the inequalities, it is paramount to understand how these characteristics influence health seeking behaviour and access to healthcare for this population. To improve overall education at a national level is a continuous process and is not achievable in a short time; rather, health programs or simple mass media communication focusing on symptom recognition targeting populations with low education could help improve awareness and early care seeking for lung cancer.

Earlier health-seeking behaviour of individuals also affected subsequent time intervals. Seeking care from multiple providers before diagnosis predicted longer durations in four intervals. It was out of the scope of the study to further investigate the reasons behind multiple consultations or the types of providers from whom care was sought. The results could reflect lack of availability of services, lack of clear referral pathways, or other issues with the healthcare system. This is an area for further study.

Characteristics of the healthcare system were important predictors in our study. Informal healthcare is deeply rooted in the health care system in Bangladesh as a part of primary care; it is more accessible and affordable for people with lower education and low incomes. Seeking care from informal care providers predicted a longer interval from diagnosis to treatment, possibly due the effect from earlier intervals. Providing training or incentivising informal care providers to be able to recognise symptoms and advise patients for proper investigation directly or through a medically trained provider could be a possible intervention to reduce delays.

This study has some limitations. It was a hospital based study and data were collected from patents at three hospitals during their inpatient care. Caution should be exercised when extrapolating our findings to other hospitals in Bangladesh as we did not investigate the potential impact of regional and geographical variation of factors on timeliness of care. Data on timepoints to determine the length of intervals were collected from patient recall and medical record review. Where patient recall was the only data source, the precision of the record of the date of the onset of symptoms may have been affected, either to under or overestimate intervals. Intervals between events taking place prior to entering formal healthcare (i.e., symptom onset and first contact with HCPs) are most likely to be affected by patient recall bias. The study sites chosen for this research did not have the capacity for thoracic cancer surgery hence the included participants only underwent chemotherapy or radiotherapy treatment. This is a limitation of the study, as we could not investigate the duration of this interval for patients who were advised to have surgery. Keeping in mind that no study participants reported having surgery as the first treatment, the length of duration of intervals from symptom to first contact with an HCP and subsequent interval durations could be shorter if the study included patients who were detected early and went for surgery. Given the circumstances and the health system of the country in which the study was conducted we cannot be certain of the implications of this limitation in the sample and further research is needed. However, compared to other studies from the Indian-subcontinent, data were collected from multiple sites and the sample size was large.

## Conclusion

Our study identified predictors that affect the timeliness of seeking and receiving care for lung cancer in different stages of the care seeking pathway. Multiple triggering symptoms, secondary school or above education, and higher monthly household income predicted shorter time periods while consulting with informal healthcare providers and with multiple healthcare providers predicted longer durations. Our study suggests that inequality in healthcare for lung cancer exists in Bangladesh and needs to be further explored in studies with a bigger sample size. The reason for the association between interval durations and seeking care from multiple healthcare providers should be explored further.

## Electronic supplementary material

Below is the link to the electronic supplementary material.

Supplementary Material 1

## Data Availability

The datasets generated and/or analysed during the current study are not publicly available because of privacy issues; data include identifiable personal health information. Data files will be available upon reasonable request to Professor Virginia Lewis and on the condition that the La Trobe University ethics committee grants permission for researchers who meet the criteria for access to confidential data. Contact information: V.Lewis@latrobe.edu.au or directly to Human Research Ethics Committee: humanethics@latrobe.edu.au.

## Abbreviations

AMCGH: Ahsania Mission Cancer & General Hospital
BDT: Bangladeshi Taka
BSMMU: Bangabandhu Sheikh Mujib Medical University
HCP: Health care provider
HIC: High Income Country
HR: Hazard Ratio
IQR: Inter Quartile Range
LMIC: Low- and Middle-Income Country
NICRH: National Institute of Cancer Research and Hospital
PH: Proportional Hazard
