# Supplementary material for: Factors influencing the timeliness of care for patients with lung cancer in Bangladesh
Source: BMC Health Serv Res. 2023 Mar 16;23:261. doi: 10.1186/s12913-023-09154-8 (PMC10018894; doi:10.1186/s12913-023-09154-8)
Supplement: Supplementary file 1 — Supplementary Material 1 [file 12913_2023_9154_MOESM1_ESM.docx]

***Model building***

In this study, we performed Cox Proportional Hazard (PH) regression analyses to assess the association of our set of independent variables with the intervals of interest.

Although stepwise regression is one of the most commonly used methods for variable selection in a regression analysis, it is heavily criticized by statisticians due to its several drawbacks such as bias in parameter estimation and inconsistency among selection algorithms [1, 2]. In this study, we used the purposeful selection method for the selection of variables to be included in the regression models. Four steps were followed to determine the final models, which are essentially identical to those used in any other regression models and widely used for Cox regression [3, 4]:

1. We began by conducting univariate analyses using nonparametric log rank tests of equality on time intervals across levels or strata of the independent variables. We then fit an initial model involving all variables which were significant in the univariate analysis at a 25% level of significance (initial models are provided in a supplementary file). The rationale for choosing a moderate level of significance is in accordance with the recommendations for linear regression [5] and for discriminant analysis [6].
2. We identified highly correlated covariates in the initial model that could be deleted based on the p-values of the Wald tests for individual coefficients. We conducted a partial likelihood ratio test to affirm that the deleted covariates were not contributing significantly to the predictive power of the regression. After fitting the reduced model containing a limited number of variables, we checked whether the exclusion of any deleted covariates induced considerable change in the coefficients of the remaining variables. If exclusion of any variable changed the regression coefficients considerably, the variable was added back into the model.
3. At this stage, we added all variables excluded, one at a time, back to the reduced model to ensure that they were not contributing significantly to the equation. Partial likelihood tests were used to assess the significance of the initially excluded variables.
4. Lastly, we examined whether interaction terms were needed in the model. Pairwise interactions were tested between all of the variables in the final model and significance of each interaction term was assessed using a partial likelihood test.

Following the steps stated above, we obtained the final model and thoroughly evaluated it using model diagnostics. The key assumption in Cox PH regression is the proportional hazard assumption. To test this assumption, scaled Schoenfeld residual tests were performed [7]. Finally, we evaluated the goodness of fit of the final models using Cox-Snell residuals.

1. Copas JB. Regression, Prediction and Shrinkage. Journal of the Royal Statistical Society Series B, Methodological. 1983;45(3):311-35.

2. Rencher AC, Pun FC. Inflation of R2 in Best Subset Regression. Technometrics. 1980;22(1):49-53.

3. Assassi S, Del Junco D, Sutter K, McNearney TA, Reveille JD, Karnavas A, et al. Clinical and genetic factors predictive of mortality in early systemic sclerosis. Arthritis Rheum. 2009;61(10):1403-11.

4. Hosmer DW, Lemeshow S, May S. Applied Survival Analysis: Regression Modeling of Time-to-Event Data. 2nd Edition ed: Wiley; 2008. 135 p.

5. Bendel RB, Afifi AA. Comparison of Stopping Rules in Forward “Stepwise” Regression. Journal of the American Statistical Association. 1977;72(357):46-53.

6. Costanza MC, Afifi AA. Comparison of Stopping Rules in Forward Stepwise Discriminant Analysis. Journal of the American Statistical Association. 1979;74(368):777-85.

7. Grambsch PM, Therneau TM. Proportional hazards tests and diagnostics based on weighted residuals. Biometrika. 1994;81(3):515-26.
